# Suicidal ideation following internet-delivered tailored CBT for depression – a secondary analysis of a factorial design trial

**DOI:** 10.3389/fpsyt.2024.1341495

**Published:** 2024-09-06

**Authors:** Anton Käll, Gerhard Andersson

**Affiliations:** ^1^ Department of Behavioral Sciences and Learning, Department of Clinical Neuroscience, Linköping University, Linköping, Sweden; ^2^ Center for Social and Affective Neuroscience, Linköping University, Linköping, Sweden; ^3^ Department of Clinical Neuroscience, Karolinska Institutet, Stockholm, Sweden

**Keywords:** iCBT, suicidal ideation, depression, internet intervention, factorial trial

## Abstract

**Introduction:**

Suicidal ideation is common in major depressive disorder (MDD) and a risk factor for suicidal behavior. Although it can be reduced with psychological treatments, the risks often make clinicians hesitant to offer treatment. This concerns remote treatment options, such as internet-delivered cognitive behavior therapy (ICBT), which may be considered unsafe. Although previous studies indicate that ICBT can reduce self-reported suicidal ideation both as primary and indirect treatment target, questions remain about under what circumstances ICBT can be offered as the primary treatment. In this secondary report, we investigated the importance of different treatment factors in reducing suicidal ideation via ICBT, including different kinds of therapist support.

**Methods:**

We analyzed data from 197 participants from a factorial trial of ICBT for symptoms of MDD. Before inclusion all participants completed a structured clinical interview where obvious suicidal intent lead to exclusion. Suicidal ideation was assessed at pre- and posttreatment using one item of the PHQ-9 and one from BDI-II. The data were analyzed using generalized linear models.

**Results:**

The pre- to posttreatment comparisons showed decreases in the reporting of suicidal ideation. Findings were consistent across the two measures that was used. There was no effect of support format and content tailoring.

**Conclusions:**

The findings suggest that ICBT can help alleviate suicidal ideation even when it is not the focus of the treatment. This was the case regardless of mode of therapist support, who tailored the treatment content, and if case supervision was available or not.

## 1 Introduction

Suicidal ideation (SI) is a common symptom among those suffering from major depressive disorder (1), and serves as a risk factor for subsequent suicide attempts (2). Because of the risks linked to SI, it has been described as an essential intervention target (3).

Psychological treatments are an option for decreasing SI and suicidal behaviors, both as a direct target and indirectly via treating other forms of psychopathology (e.g., major depressive disorder). Méndez-Bustos et al. (4) noted that a range of different psychotherapeutic approaches have been used to reduce SI and other suicidal outcomes, including Cognitive Behavior Therapy (CBT), Dialectal Behavior Therapy (DBT), and Interpersonal Psychotherapy (IPT). CBT is based on altering behaviors and one’s stance towards thoughts and internal sensations as a means of treating psychopathology (5). In the treatment of SI, CBT relies on strategies such as challenging biased thinking and suicidal cognitions, as well as behavioral strategies such as relaxation techniques (6). A later umbrella review (7) reported a significant, albeit small, average reduction in SI following CBT with the authors mentioning scalability as an important advantage. However, some questions remain. For example, Mewton and Andrews (6) noted that there is still insufficient evidence that CBT reduces SI when it serves as an indirect target. Because of the inconclusive evidence up to this point, additional studies are needed.

Another consideration when treating SI is how the treatment can be delivered in an effective, safe, and accessible manner. Internet-delivered cognitive behavioral therapy (ICBT) can be useful for this purpose as it is more easily accessible than alternative contact modes, can be offered anonymously, and can reduce concerns about stigma associated with SI (8). ICBT is a form of self-help, with psychoeducation and practical strategies delivered via a web platform or an app (9). The treatment content is often delivered in a modular fashion, much like how regular CBT sessions cover different topics and assignments over the course of a treatment. It is frequently delivered with asynchronous guidance from a clinician. Among the advantages are that ICBT and internet interventions in general can reduce costs and bridge distances to specialist clinics. However, ICBT also comes with challenges, such as the lack of face-to-face contact with a clinician. Due to this, clinicians may be reluctant to offer ICBT in the presence of SI, despite the support for ICBT when treating several mental and somatic health problems (10). More specifically, internet-delivered cognitive behavioral therapy (ICBT) is effective for reducing symptoms of depression (11), and can be as effective as face-to-face CBT (12). In relation to parasuicidal outcomes, ICBT has been found to produce small but statistically significant reductions in suicidal ideation when it serves as the primary target for the treatment (13). Importantly, results of some trials investigating ICBT for depression have reported reductions in SI, suggesting that the treatment does not need to target SI directly (14, 15). Rather, in cases with less severe and persistent forms of SI, ICBT could be a viable treatment option that addresses both SI and other symptoms of depression. However, not all studies show secondary benefits of ICBT for depression on measures of SI. Helen et al. (16) did not find within-group reductions of SI following ICBT, regardless of whether the participants had received therapist support or not. Additional studies would be helpful to help clarify the effects of ICBT for SI, in particular when SI is not the primary treatment target.

Another consideration is whether there are factors related the treatment that can improve the effects and make it more suitable for certain populations. One example is the presence of weekly therapist support that has been linked to better treatment outcomes and better adherence compared to not having such support available on a weekly basis (17). Another example from an open study and two factorial design trials is the choice of treatment content and allowing participants to choose the content themselves compared against therapists making the choice (18–20). Investigations into factors such as these could be of importance from a decision-making standpoint as they offer information about what kind of treatment structure that offers the best chance of improvement for people seeking help.

The current study sought to expand on previous studies investigating the efficacy of internet-delivered CBT for depression in reducing suicidal ideation. More specifically, the primary aim of this secondary report was to investigate both the general effects of ICBT on SI, but also other factors relevant to the effects of ICBT for SI. These included the kind of therapist-guidance that was offered, decisions on treatment content, and the availability for case supervision for therapists guiding the participants through the treatment. Based on prior studies of similar treatments, it was hypothesized that suicidal ideation would decrease overall during the treatment. For the treatment factors, we wanted to explore whether the on-demand approach to guidance produced similar reductions in SI to the standard way of providing weekly guidance that is often used in regular care settings. Likewise, we were interested in whether participants receiving the option to tailor their own treatment had similar reductions in SI compared to the usual way of having the therapist tailor the content of the intervention. Lastly, we were interested in whether the availability of supervision for the therapists was important for the treatment’s ability to reduce SI. The analyses of treatment factors were conducted in an exploratory manner with no directional hypotheses regarding which condition that would perform better.

## 2 Materials and methods

The current study was a secondary analysis of a trial that has been described in detail in a previous article (19). The project received ethical approval from the regional ethics board. All participants provided informed consent before registering for the study.

### 2.1 Design

The trial was a randomized factorial trial in which the included participants were randomly assigned to one of eight conditions based on three different factors/independent variables. First, participant could receive either regular, once-weekly therapist support or have the option to request therapist support (referred to as on-demand support). Second, participants could either have the content of the intervention tailored for them by a therapist, or tailor it themselves. Third, the therapist assigned to the participant could either be eligible to receive supervision for the case or not. All participants began treatment at the same time but had different combinations of the independent variables in a balanced factorial design.

The treatment lasted for 10 weeks. During this time, participants received access to modules covering psychoeducation and strategies for dealing with depression and comorbid anxiety disorders (e.g., panic disorder), and transdiagnostic problems (e.g., perfectionism). The full content of the program is described in the original article (19). Treatment modules included exercises specific to depression (e.g., behavioral activation), more generic and transdiagnostic CBT principles (e.g., cognitive restructuring, gradual exposure, applied relaxation), and problem-specific treatment components (e.g., insomnia, stress). None of the modules dealt with suicidal ideation explicitly. Participants in the therapist-tailored condition received eight modules on average, while those with self-tailored content could select as many or as few modules as they wanted (but were instructed that eight modules was the average pace).

### 2.2 Recruitment, inclusion, and exclusion criteria

**Figure 1:**
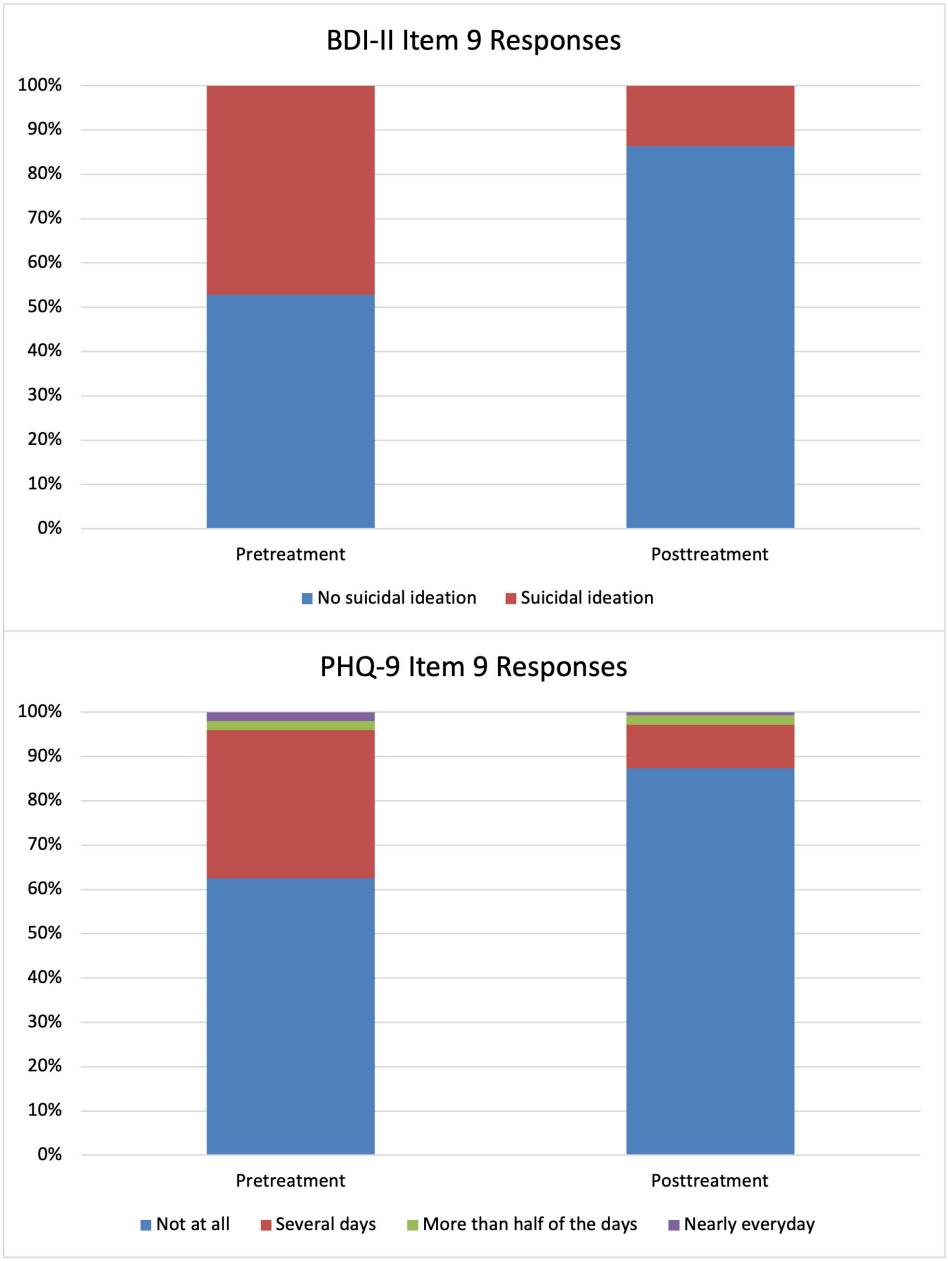
Proportion of Participants with Suicidal Ideation Before and After Treatment.

Detailed information about the recruitment procedure is presented elsewhere (19). Briefly, trial recruitment took place via multiple sources, including via social media posts and posters in primary care settings around Sweden. The study information specified that we were looking to recruit participants that experienced low mood and other symptoms of major depressive disorder. Prospective participants would register on the study website and fill in a screening. They would then complete a structured clinical interview (MINI 7.0; 21) before a decision on inclusion/exclusion was made. The inclusion criteria stated that the participant had to 1) be 18 years old or older, 2) meet the criteria for major depressive disorder or unspecified depressive disorder, 3) have elevated symptoms of major depressive disorder on the Patient Health Questionnaire-9 (PHQ-9; a sum score of at least 5) and/or Beck Depression Inventory, version two (BDI-II; a sum score of at least 10), 4) have access to the internet and a device with a web browser, 5) sufficient proficiency in Swedish, 6) no other ongoing psychotherapy and, if using psychotropic medication, a stable dose for the past three months. Participants were excluded if they a) had an active substance use problem, b) had severe psychiatric problems (e.g., anorexia nervosa) that could not be managed within the framework of the study, or c) expressed suicidal plans or preparations. The last criterion was assessed using the questions regarding suicidal ideation and suicide on the MINI 7.0. In total, seven participants were excluded for this reason. A total of 197 participants were included. All included participants were randomized by an independent party not involved in other parts of the project. A complete flowchart of the recruitment process can be seen in **Figure 1** of the article reporting on the primary outcomes (19).

### 2.3 Outcome measures

Two items were used to assess the prevalence and frequency of suicidal ideation. First, we used item nine from the Patient Health Questionnaire (PHQ-9; 22) which asks the respondent to indicate how often they have been bothered by “thoughts that you would be better off dead or of hurting yourself in some way?”. Responses are given on a four-point scale with the alternatives being Not at all (scored as 0), Several days (1), More than half of the days (2), and Nearly every day (3). Second, we also used an item from the second iteration of the Beck depression inventory (BDI-II; 23) which asks the participant to indicate the prevalence and severity of suicidal ideation and suicidal intent. The responses given are either I do not have any thoughts of killing myself (scored as 0), I have thoughts of killing myself, but I would not carry them out (1), I would like to kill myself (2), and I would like to kill myself if I had the chance (3). Both measures were administered before and after the active treatment phase.

### 2.4 Statistical analysis

Statistical analyses were conducted using R (24). The intention-to-treat was used across all analyses. An alpha level of.05 was used for statistical inferences and 95% confidence intervals are reported. A Fisher’s exact test was used to test the relationship between the presence of suicidal ideation at the pre-treatment timepoint and the likelihood of having missing data at the posttreatment timepoint.

To assess the change in suicidal ideation during the treatment phase we specified two generalized linear models using the *glm* function in R. For each model, we were interested in the change over time (from pre-treatment to posttreatment), and the interaction between time and each of the three factors outlined above (who tailored the treatment, which kind of support that was provided, and if case supervision was allowed for the specific participant). The factors were coded as: -0.5 = therapist-tailored content, 0.5 = self-tailored content, -0.5 = scheduled therapist support, 0.5 = on-demand support, -0.5 = case supervision available, 0.5 = case supervision not available. The models also estimated the higher order interactions. With the non-normal distribution of responses in mind, we specified a logistical regression using the binomial family argument. For the responses on item 9 of PHQ-9, we recoded the few responses with a score of 2 and 3 as a score of 1, thus creating a dichotomous outcome for use in a logistical regression analysis. For item 9 of the BDI-II, the responses were already exclusively scored as 0 or 1, meaning that no recoding was necessary. Across both outcome measures, a score of 0 indicate no suicidal ideation and 1 indicate the presence of suicidal ideation. In addition to these main analyses, we also explored whether gender moderated the general treatment effect (via a time * gender interaction). For these analyses, gender was coded as -0.5 = woman, 0.5 = man.

## 3 Results

The demographics of the sample are described in **Table 1**. Overall outcomes from the trial have been summarized in Andersson et al. (19). Missing data at the posttreatment timepoint was not significantly related to the presence of suicidal ideation at pre-treatment, Fisher’s exact *p* = .211 for item 9 on the BDI-II and .626 for item 9 on the PHQ-9.

**Table 1:** Demographic characteristics of the sample (*n* = 197).

	*M*	SD
Age	34.59	13.19
Gender	*n*	%
Female	152	77.2
Male	44	22.3
Other	1	0.5
Educational level		
Primary school	3	1.5
High school	28	14.2
Vocational education	17	8.6
University (ongoing)	63	32
University (finished)	86	43.7
Current psychotropic medication: *% yes*	30	15.2
Prior psychological treatment: *% yes*	105	53.3
Somatic illness: *% yes*	10	5.1

### 3.1 Prevalence and change in suicidal ideation

The percentages of participant indicating that they experienced suicidal ideation according to the items of PHQ-9 and BDI-II can be seen in **Table 2**. Estimates from the generalized linear models are available in **Table 3**. The prevalence of suicidal ideation in the sample and the change over time is visualized in **Figure 1**. Both the observed percentages and the model coefficients indicated a reduction in suicidal ideation during the treatment phase. The odds ratios for both outcome measures showed a significantly reduced risk of experiencing suicidal ideation at the end of the treatment compared to before the start of the study. For both the BDI-II item and the PHQ-9 item, neither of the three factors predicted an increase or decrease in suicidal ideation. None of the higher order interactions were statistically significant.

**Table 2:** Percentages of participants in the sample with suicidal ideation at the pre- and posttreatment treatment timepoints.

	Pretreatment	Posttreatment
PHQ-9 Item 9	% (n)	% (n)
Not at all	62.4 (123)	87.3 (124)
Several days	33.5 (66)	9.9 (14)
More than half of the days	2 (4)	2.1 (3)
Nearly everyday	2 (4)	0.7 (1)
BDI-II Item 9	% (n)	%
I do not have any thoughts of killing myself	52.8 (104)	86.3 (120)
I have thoughts of killing myself, but I would not carry them out	47.2 (93)	13.7 (19)

PHQ-9, Patient Health Questionnaire 9; BDI II, Beck Depression Inventory II.

**Table 3:** Coefficients and odds ratios for the generalized linear models.

Outcome	Coeff.	95% CI	SE	*p*-value	OR	OR 95% CI
BDI-II Item 9						
Time	-1.98	-2.71, -1.36	0.34	<.001	0.14	0.01, 0.37
Time X Choice	-0.68	-2.08, 0.63	0.67	.312	0.50	0.12, 1.88
Time X Support	0.00	-1.39, 1.32	0.67	1	1.00	0.25, 3.75
Time X Supervision	0.00	-1.36, 1.36	0.67	1	1.00	0.26, 3.88
PHQ-9 Item 9						
Time	-1.62	-2.35, -1.00	0.34	<.001	0.20	0.10, 0.37
Time X Choice	-0.50	-1.89, 0.81	0.67	.458	0.61	0.15, 2.25
Time X Support	0.29	-1.07, 1.65	0.67	.668	1.34	0.34, 5.18
Time X Supervision	-0.11	-1.47, 1.25	0.67	.874	0.90	0.23, 3.50

### 3.2 Gender differences for changes in suicidal ideation

The results from the exploratory analyses indicated that the change over time in SI did not differ between women and men according to the BDI-II item, estimate = 0.91 [95% CI -0.34, 2.11], SE = 0.62, *p* = .145, OR = 2.47 [95% CI 0.73, 8.35]. The same was true for the PHQ-9 item, estimate = 0.95 [95% CI -0.35, 2.20], SE = 0.65, *p* = .141, OR = 2.58 [95% CI 0.73, 9.14].

## 4 Discussion

The results in this study are in line with previous trials indicating that suicidal ideation decrease following treatment with CBT more generally (25), and ICBT more specifically (14, 15). The findings from this factorial design trial support the idea that less severe SI can be treated within the context of a more generic version of ICBT for depression without the need for elements tailored specifically for SI. This finding is promising as ICBT has been suggested to fit some important needs of people burdened by SI, including the potential for anonymity and widespread availability (8). It also serves as an additional indication of the usefulness of non-SI specific treatments for reducing SI, which is an approach to this problem for which more evidence is needed (6). It should be noted that severe SI did serve as an exclusion criteria, which means that the potential of the treatment to help populations with more severe symptoms remains unknown. However, in populations with less severe depressive symptoms and SI, the findings support the use of ICBT.

We found no difference in the reductions between participants who received scheduled support versus those who had support on-demand. This is important as it shows that the efficacy of ICBT in reducing is SI may not be dependent a specific support format and that the treatment can be effective when delivered with less therapist-intense forms of guidance. The latter point is reminiscent of the findings reported by Watts et al. (15), who also reported decreases in SI while not utilizing weekly, regular therapist support. The findings from our present study could also be taken as an additional indication of the utility of the on-demand support format in general, which has also been found in a study on social anxiety disorder (26) and in transdiagnostic treatments for symptoms of anxiety and depression (27). Given the comparable efficacy and the additional time-efficiency relative to weekly, regular scheduled support, support on-demand could be of importance in future efforts of implementing and disseminating similar types of treatment.

The lack of a difference in the efficacy between self-tailored and therapist-tailored module content differs from the small but statistically significant effect reported in the original article on BDI-II ratings (19). Overall, this difference could not be expected to be relevant for SI as a module on SI was not included. The reduction of SI in our sample is comparable to other similar interventions that also primarily focus on reducing depressive symptoms, rather than focusing on SI (15). An aspect to investigate in future studies is whether the addition of a module focused specifically on SI could further improve the effects of the intervention.

### 4.1 Strengths and limitations

The strengths of the study include the use of two outcome measures for measuring suicidal ideation, which allows for more robust conclusions on the reductions in SI. However, the findings should also be interpreted with the methodological limitations in mind. First, although participants were randomized to different conditions, neither of these served as a no treatment/placebo control group. This limits the causal conclusions about the impact of the treatment. The lack of an untreated group to compare against means that we cannot rule out that the observed change in SI was due to factors that did not relate to the treatment, such as a natural decline over time during the treatment period. Other potential explanations include regression to the mean, where extreme values are less likely to occur with repeated sampling which could cause the illusion of a symptom decrease. Finally, the lack of an active control group that receives access to the general treatment procedure also makes it more difficult to conclude that it was the CBT content that reduced SI, and no other non-treatment factors such as contact with a therapist or the measurements procedures during the study. The fact that we did not observe a difference between those that that received weekly support and those that received support on-demand (which was asked for infrequently) do suggest that the support itself might not have been the important ingredient, but other factors could still have played a role. Second, the exclusion criteria also limit the generalizability of the findings as too severe suicidal ideation would have served as an exclusion criterion. Indeed, seven participants in the original trial were excluded due to suicidal intentions (details provided in 19), and it is unknown whether these individuals would have experienced the same reductions in SI during the treatment that we saw for the included participants. Because of this, the conclusions from the present study may not generalize to populations with more severe forms of SI, including plans for suicide.

## 5 Conclusions

Overall, the results of the trial are in line with previous studies that suggests that ICBT for depressive symptoms reduces the self-reported prevalence of suicidal ideation. This finding supports the notion that less frequent and severe forms of suicidal ideation can be treated within the context of ICBT and should not serve as an exclusion criterion for this kind of treatment. The study also adds to the field by providing an additional indication that this decrease in suicidal ideation is not contingent on scheduled therapist support, but that reductions also occur when the support is provided on-demand. This is encouraging from an implementation standpoint as this support format requires less time, thus potentially allowing for the rolling out of similar treatments even with a scarcity of trained therapists. Neither of the other treatment factors exhibited a significant difference, further supporting the potential for flexible applications of the treatment format.

## Data Availability

The original contributions presented in the study are included in the article/supplementary materials, further inquiries can be directed to the corresponding author/s.
